# Synthesis, characterization, and biological evaluation of new copper complexes of naphthyl pyrazole ligands

**DOI:** 10.3906/kim-2010-5

**Published:** 2021-06-30

**Authors:** Melek HINIS, Kuldip SINGH, Demet ERDÖNMEZ, Ayfer MENTEŞ

**Affiliations:** 1 Department of Chemistry, Faculty of Arts and Sciences, Aksaray University, Aksaray Turkey; 2 Department of Chemistry, Leicester University, Leicester, England UK; 3 Department of Biology, Faculty of Arts and Sciences, Aksaray University, Aksaray Turkey

**Keywords:** Naphthyl, pyrazole, copper, antimicrobial activity

## Abstract

Two naphthalene pyrazole ligands were synthesized using KOH/DMSO and Cu catalyst and characterized with FT-IR, ESI-MS, ^1^H, and ^13^C NMR spectroscopies. The crystal structures of 1-(2-methylnaphthalen-1-yl)-1H-pyrazole (MeNap-Pz) ligand have been determined with X-ray crystal structure analysis. Reaction of the ligands with Cu(NO_3_)_2_x3.5H_2_O gave two new complexes and characterized with magnetic susceptibility, molar conductance, FT-IR, LCMS-MS, ICP-OES, NMR, thermogravimetric analysis, and ESR spectra. The spectral data of the ligands are coordinated to the metal ion through the nitrogen atoms of the pyrazole ring. Consequently, it has been determined that [Cu(MeNap-Pz)_2_(NO_3_)]NO_3_.2H_2_O complex showed square planar geometry and [Cu(NapMe-Pz)_2_(NO_3_)_2_].H_2_O complex showed octahedral geometry. All compounds were screened for in vitro antibacterial activity and copper complexes have been shown to be effective on bacteria.

## 1. Introduction

Pyrazole type ligands are very important in organometallic chemistry due to their wide usage area. Pyrazoles are aromatic ring organic compounds of the heterocyclic series with a 5-membered ring structure having three carbon and two nitrogen atoms [1–3]. Pyrazole derivatives exhibit many biological and pharmacological properties such as antibacterial, antiinflammatory, anticancer, antiviral, fungicidal, herbicidal, pesticide, and other biological activities [4–9]. In recent studies, it has been determined that naphthyl pyrazole ligands, which are pyrazole derivatives, and their metal complexes are also biologically active [10–12]. Pyrazole derivatives have also been used as ligands for formation of iridium complexes in organic light emitting diode (OLED) materials [13]. 

Due to the presence of two nucleophilic centers in the pyrazole ligands, the pyrazole nucleus being thermally and hydrolytically stable, the ability to coordinate in many ways and the pyrazole ligand to make hydrogen bonds, a wide variety of inorganic structures can be produced [14,15].

N-Aryl pyrazoles have been synthesized by reaction of 1,3-diketones with aryl hydrazines for many years. In accordance with the nature of the 1,3-diketones, undesirable isomers may be formed in such reactions [16]. In recent years, many methods that achieve heterocyclic N-arylation using catalytic Pd and Cu reactions have been reported [17]. Although the conditions in the N-arylation of the heterocyclic ligands using the Pd catalyst were mild [18], significant progress was made in the Cu-catalyzed N-arylation of pyrazoles due to both the high price of the Pd catalyst and the inadequate ligand concentration for the heterocyclic substrates [19,20]. For the direct arylation of pyrazole, bases such as KOH, KOt-Bu, or NaH are used in polar aprotic solvents such as DMSO, DMF [21–24].

Copper complexes of pyrazole are widely used. It is preferred in the synthesis of complexes because copper is easily available and insensitive to light or air, and it is the most economical way to form C−C bonds, which are increasingly important in both industrial and academic fields. Copper pyrazole complexes form thermally stable structures, and are used as effective catalysts in many reactions and biologically active structures are formed in the literature [25–27].

In this study, 1-(2-methylnaphthalen-1-yl)-lH-pyrazole (MeNap-Pz) and 1-(naphthalen-2-ylmethyl)-lH-pyrazole (NapMe-Pz) ligands were synthesized by the reaction of pyrazole with 1-bromo-2-methylnaphthalene and 2-(bromomethyl) naphthalene in the KOH/DMSO system using Cu catalyst. Two novel copper(II) complexes with these ligands were obtained and characterized with FT-IR, NMR, and ESI-MS. The X-ray diffraction structures of the compound 1-(2-methylnaphthalen-1-yl)-l *H* -pyrazole (MeNap-Pz) were determined. The synthesized copper complexes were characterized with magnetic susceptibility, molar conductivity, FT-IR, NMR, LC MS-MS, ICP-OES, TGA, UV-Vis, and ESR spectra. All the synthesized compounds were evaluated for their antibacterial properties against various pathogenic bacterial strains ( *Staphylococcus aureus* , *Pseudomonas aeruginosa* , *Klebsiella pneumoniae* , *and Bacillus cereus* ) using the minimum inhibitory concentration method. 

## 2. Materials and methods

All chemicals were purchased from Merck or Aldrich and were used without further purification. All solvents were dried and kept over molecular sieves prior to use.

The infrared spectra (4000–650 cm^–1^) were recorded on a PerkinElmer Spectrum 100 FT-IR Spectrophotometer (PerkinElmer, Inc., Waltham, MA, USA) using the ATR technique. ^1^H and ^13^C-NMR spectra were measured on a Bruker DRX 400 MHz (Bruker, Corp., Billerica, MA, USA) or Agilent 600 MHz nuclear magnetic resonance instrument (Agilent Technologies, Inc., Santa Clara, CA, USA) (CDCl_3_ and DMSO-d_6_ as solvent).

Low-resolution electrospray ionization mass spectra (ESI-MS) were obtained on a micromass Quattro LC mass spectrometer (Waters, Corp., Milford, MA, USA) in acetonitrile or methanol as HPLC grade. Mass spectra LC-MS/MS were measured on a Thermo TSQ Quantum Access Max spectrometer (Thermo Scientific, Waltham, MA, USA). Copper contents of the complexes were obtained with PerkinElmer Optima 2100 DV inductively coupled plasma (ICP) optical emission spectrometry (OES).

Melting points were determined using Büchi B-540 brand melting point apparatus.

Thermogravimetric analyses (TGA) were performed under N_2_ atmosphere at 1 atm with a heating rate of 10 °C/min on a PerkinElmer EXSTAR S11 7300 TG/DTA.

The ESR spectra were recorded with Jeol Jesfa-300 ESR X-band spectrometer using 9.5 GHz modulation.

UV-VIS spectra of the compounds were taken between 190 and 1000 nm using Genesys 10S UV-VIS spectrophotometer. Conductivities of solutions (c = 1.0 × 10^–3^ M in methanol) were measured in a 25 °C temperature with Table Top CD-2005 brand conductometer. Magnetic susceptibility of complexes was measured in a MK1 Sherwood Scientific magnetic susceptibility apparatus.

### 2.1. Synthesis of ligands

#### 2.1.1. General comments

The materials used in the syntheses were all commercially available and were used without purification. All solvents were dried and kept over molecular sieves prior to use.

Although 1-(Naphthalen-2-ylmethyl)-1H-pyrazole (NapMe-Pz) ligand was synthesized by the cross-coupling reaction of aryl halides [28] and 1-(2-Methylnaphthalen-1-yl)-1H-pyrazole (MeNap-Pz) ligand was synthesized by microwave-assisted, cuprous oxide catalyzed coupling of aryl halides with pyrazole reaction [29] in previous studies, MeNap-Pz and NapMe-Pz ligands were synthesized using a modified method which includes the reaction of pyrazole with 1-bromo-2-methylnaphthalene or 2-(bromomethyl) naphthalene in the KOH/DMSO system using Cu catalyst for the synthesis of the ligands [30,31].

##### 2.1.1.1. Synthesis of 1-(2-Methylnaphthalen-1-yl)-1H-pyrazole, (MeNap-Pz)

**Scheme 1:**
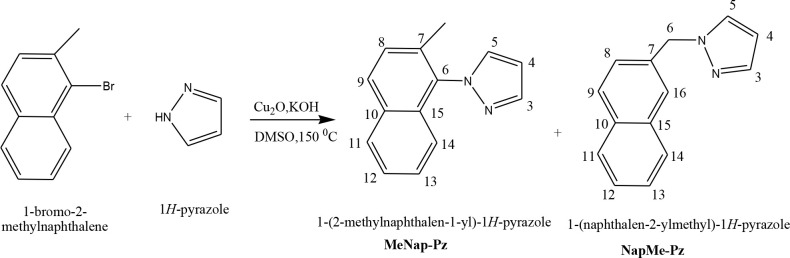
MeNap-Pz and NapMe-Pz ligands synthesis scheme.

1-Bromo-2-methylnaphthalene (2.455 g, 10 mmol), pyrazole (1.090 g, 28 mmol), copper(I) oxide (0.150 g, 10 mmol), and KOH (1.15 g, 20 mmol) were stirred for 48 h in dried DMSO (20 mL) at 150 °C under a nitrogen atmosphere. The resulting black heterogeneous mixture was cooled to room temperature. Dichloromethane was added to the mixture and the mixture was filtered through silica to remove the pyrazole which did not enter the reaction. The solution was washed with water (4 × 25 mL) and dried with anhydrous MgSO_4_. The solvent was removed on the rotary evaporator to obtain the crude product from the filtrate. TLC and ^1^H NMR showed that the product was not pure; it was a mixture of MeNap-Pz and NapMe-Pz. The residue was purified with dichloromethane/petroleum ether (1:1) mixture by column chromatography from silica gel. Colorless NapMe-Pz crystals were obtained by recrystallization in CH_2_Cl_2 _and colorless MeNap-Pz crystals were obtained by recrystallization in petroleum ether. It was obtained as MeNap-Pz (Yield: 0.475 g, 22%; M.P. 62–74 °C) and NapMe-Pz (Yield: 0.277 g, 13%; M.P. 85–89 °C) (Scheme 1).

**MeNap-Pz;**

ES-MS ( *m/z)* : 208 [M], 192 [M-CH_3_], 180 [M-HCN] (100%)

FT-IR (ATR, cm^–1^): 3127-3047 u(C-H)_ar_, 2922 u(C-H)_al_, 1633 u(C=N), 1599–1507 u(C=C), 1370 u(C-N), 1040 u(N-N). 

^1^H NMR: (400 MHz, CDCl_3_), δ (ppm): 7.87–7.83 (3H, m, H_14_, H_11_, H_9_), 7.61 (1H, dd, H_3, _J =2.3 Hz), 7.46–7.39 (3H, m, H_12_, H_13_, H_5_), 7.12 (1H, dd, H_8_, J = 8.0 Hz), 6.55 (1H, t, H_4_, J = 2.1 Hz), 2.21 (3H, s, CH_3_). 

^13^C NMR (CDCl_3_, 100.6 MHz), δ (ppm): 140.46 (C_3_), 133.72 (C_6_), 132.51 (2C; C_10_, C_15_), 131.55 (C_11_), 129.12 (C_14_), 128.31 (C_7_), 127.64 (2C; C_5_, C_8_), 127.31 (C_13_), 125.72 (C_12_), 122.53 (C_9_), 106.09 (C_4_), 17.56 (CH_3_).

##### 2.1.1.2. Synthesis of 1-(Naphthalen-2-ylmethyl)-1H-pyrazole, (NapMe-Pz)

**Scheme 2:**
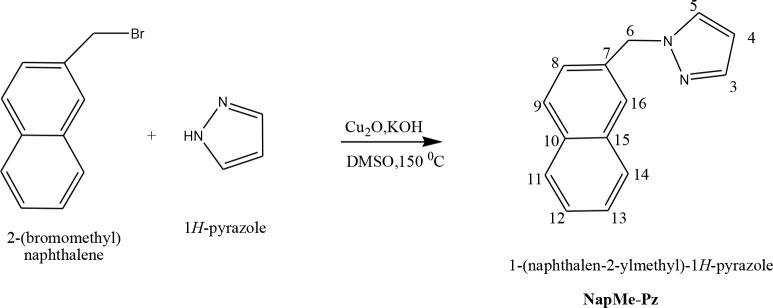
NapMe-Pz synthesis scheme.

The 1-(Naphthalen-2-ylmethyl)-1 *H* -pyrazole was synthesized and isolated with the same method as MeNap-Pz, using 2-(bromomethyl)naphthalene (0.982 g, 4 mmol) and pyrazole (0.436 g, 6.4 mmol) stirring for 48 h in dried DMSO (20 mL) at 150 °C under a nitrogen atmosphere. TLC and ^1^H NMR results showed that the product was pure. It was obtained as colorless NapMe-Pz crystals (Yield: 0.453 g, 54 %; M.P. 85–89 °C) (Scheme 2). The analytical results of NapMe-Pz were determined to be the same as the compound in the first synthesized mixture in Scheme 1. 

**NapMe-Pz;**

ES-MS ( *m/z)* : 207 [M^-^], 180 [M-Pz] (100%)

FT-IR (ATR, cm^–1^): 3130-3049 u(C-H)_ar_, 2970 u(C-H)_al_, 1737 u(C=N), 1600–1509 u(C=C), 1370 u(C-N), 1047 u(N-N).

^1^H NMR: (400 MHz, CDCl_3_), δ (ppm): 7.78 (3H, m, H_9_, H_11_, H_14_), 7. 63 (1H, s, H_16_), 7.56 (1H, dd, H_3, _J = 1.5 Hz), 7.45 (2H, m, H_12_, H_13_), 7.37 (1H, d, H_5_, J = 2.6 Hz), 7.30 (1H, dd, H_8_, J = 8.2 Hz, J = 1.8 Hz), 6.27 (1H, t, H_4_, J = 2.0 Hz), 5.44 (2H, s, H_6_).

^13^C NMR (CDCl_3_, 100.6 MHz), δ (ppm): 139.57 (C_3_), 134.06 (C_7_), 133.31 (C_15_), 132.97 (C_10_), 129.25 (C_5_), 128.68 (C_8_), 127.91 (C_11_), 127.69 (C_14_), 126.69 (C_9_), 126.36 (C_16_), 126.21 (C_13_), 125.39 (C_12_), 106.03 (C_4_), 56.09 (C_6_).

### 2.2. Synthesis of complexes

#### 2.2.1. Synthesis of [Cu(MeNap-Pz)_2_(NO_3_)]NO_3_.2H_2_O complex

**Scheme 3:**
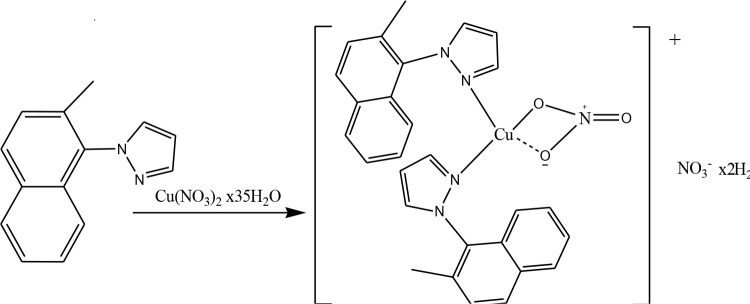
The synthesis scheme of the [Cu(MeNap-Pz)2(NO3)]NO3.2H2O complex.

1 mmol (0.208 g) of MeNap-Pz dissolved in acetonitrile (8 mL) was added to a solution of Cu(NO_3_)_2_x3.5H_2_O (1 mmol, 0.250 g) in acetonitrile (20 mL). The mixture was heated to reflux for 4 h and its color turned to green. TLC showed that the complex was formed. After evaporation, the green solid was filtered off, and washed with diethyl ether and dried at room temperature (DTG_mak_: 220 °C, M/L: 1/1, Yield: 0.333 g, 52 %). Purity of the complex was checked by TLC (Scheme 3).

LC-MS/MS ( *m/z)* : 541 [ML_2_NO_3_]^+^, ICP-OES wt% of Cu, found (Calc.% *)* : 11.90 (10.21).

L_M_: 129 Ω^–1^ cm^2^ mol^–1^.

FT-IR (ATR, cm^–1^): 3545 u(O-H; H_2_O), 3128–3054 u(C-H)_ar_, 2916 u(C-H)_al_, 1635 u(C=N), 1601–1577 u(C=C), 1373 u(C-N), 1062 u(N-N), 1480, 1277, 1011 u(N-O, NO_3_).

^1^H-NMR (600 MHz, DMSO-d_6_), δ (ppm): 7.9–6.9 (br, H-Ar), 6.5 (H_4_-Pz), 2.0 (H-CH_3_). 

^13^C-NMR (150 MHz, DMSO- d_6_), δ (ppm): 133.5–122.4 (C-Ar), 140.7 (C_3_-Pz), 135.4 (C_5_-Pz), 106.9 (C_4_-Pz), 17.5 (CH_3_).

#### 2.2.2. Synthesis of [Cu(NapMe-Pz)2(NO3)2].H2O complex

**Scheme 4:**
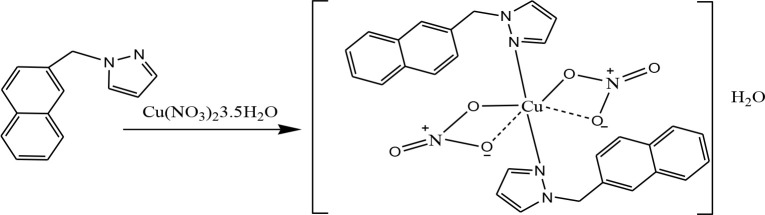
The synthesis scheme of the [Cu(NapMe-Pz)_2_(NO_3_)_2_].H_2_O complex.

0.2 mmol (0.04 g) of NapMe-Pz dissolved in acetonitrile (5 mL) was added to a solution of Cu(NO_3_)_2_x3.5H_2_O (0.06 mmol, 0.016 g) in acetonitrile (4 mL). The mixture was heated to reflux for 5 h and its color turned from brown to green. TLC showed that the complex was formed. After evaporation, the green jelly-like substance was obtained. The complex was washed with ethanol and dried at room temperature (DTG_mak_: 242 °C, Yield: 0.249 g, 40 %). Purity of the complex was checked with TLC (Scheme 4).

LC-MS/MS ( *m/z)* : 639 [ML_2_NO_3_]^+^,, ICP-OES wt % of Cu, found (Calc.% *)* : 9.34 (9.92).

L_M_: 13 Ω^–1^ cm^2^ mol^–1^.

FT-IR (ATR, cm^–1^): 3409 u(O-H, H_2_O), 3132–3050 u(C-H)_ar,_, 2972–2947 u(C-H)_al_, 1633 u(C=N), 1602 u(C=C), 1396 u(C-N), 1049 u(N-N), 1483, 1275, 1010 u(N-O, NO_3_).

### 2.3. X-ray crystal structure determination

Colorless crystals of MeNap-Pz for X-Ray diffraction experiments were obtained with recrystallization in petroleum ether. Details of the structure determinations of MeNap-Pz are summarized in Table 1. Data were collected on a Bruker Apex 2000 CCD diffractometer using graphite-monochromated Mo KR radiation, λ = 0.7107 Å at 150 K. The data were corrected for Lorentz and polarization effects, and empirical absorption corrections (SADABS) [32] were applied in all cases. The structures were solved using Patterson methods and refined with full-matrix least-squares on F^2^ using the program SHELXTL [33]. All hydrogen atoms bonded to carbon were included in calculated positions (C-H = 0.96 Å) using a riding model. Figures were drawn using the program ORTEP [34]. All nonhydrogen atoms were refined with anisotropic displacement parameters without positional restraints.

**Table 1:** Crystal data and structure refinement for MeNap-Pz

Empirical formula	C14H12N2
Formula weight	208.26
Temperature, K	150(2)
Wavelength, Å	0.71073
Crystal system	Triclinic
Space group	P-1
a, Å	7.589(2)
b, Å	8.060(2)
c, Å	9.040(3)
a, deg	77.908(5)
b, deg	85.503(5)
g, deg	84.705(5)
Volume, Å3	537.4(3)
Z	2
Density (calculated), Mg/ m3	1.287
Absorption coefficient, mm-1	0.077
F(000)	220
Crystal size, mm3	0.34 x 0.32 x 0.24
Theta range for data collection	2.31 to 24.97°.
Index ranges	-9<=h<=9, -9<=k<=9, -10<=l<=10
Reflections collected	3886
Independent reflections	1877 [R(int) = 0.0380]
Completeness to theta = 24.97°	99.0 %
Absorption correction	Empirical
Max. and min. transmission	0.969 and 0.517
Refinement method	Full-matrix least-squares on F2
Data / restraints / parameters	1877 / 0 / 146
Goodness-of-fit on F2	1.087
Final R indices [I>2sigma(I)]	R1 = 0.0588, wR2 = 0.1533
R indices (all data)	R1 = 0.0729, wR2 = 0.1618
Largest diff. peak and hole	0.223 and -0.208 e.Å-3

Atomic coordinates, bond lengths and angles, and thermal parameters have been deposited at the Cambridge Crystallographic Data Centre, CCDC Nos. 1944106.

### 2.4. Antibacterial activity

All the synthesized ligands and their corresponding Cu(II) complexes were screened in vitro for their biological activity by using two gram-positive bacteria, namely *Staphylococcus aureus* ATCC 29213, *Bacillus cereus* ATCC 10876 and two gram-negative bacteria, namely *Pseudomonas aeruginosa* ATCC 27853 and *Klebsiella pneumoniae* ATCC 700603, which caused widespread hospital infections and severe antibiotic resistance. Well diffusion method was used in antimicrobial activity trials. After 100 µL of previously produced microorganism culture was adjusted according to McFarland 0.5 (1.5 × 10^8^ CFU/mL) turbidity, it was inoculated on the surface of Mueller-Hinton agar by sterile swab spreading. Under sterile conditions, 5 mm diameter wells were dug in the agar media using a cork borer. Ligands and copper complexes were prepared at a concentration of 90 mg/mL, 20 µL was added to the individual wells and the culture was incubated for 24 h at 37 °C. Standard antibacterial drug (gentamycine) was screened under similar conditions for comparison. Activity was determined at the end of 24 h by measuring the diameter of the zone showing complete inhibition (mm) and the experiments were performed in 3 replications [35,36]. 

### 2.5. Minimum inhibitory concentration (MIC)

It was determined using the microdilution method according to procedures developed by the National Committee of Clinical Laboratory Standards [37]. The minimum inhibitory concentration was determined by assaying at 90 mg/mL, 45 mg/mL, 22.5 mg/mL, 11.25 mg/mL, and 5.625 mg/mL concentrations along with standards at the same concentrations. The lowest compound concentration inhibiting visible bacterial growth is reported as MIC. The experiment was repeated 3 times.

## 3. Result and discussion

The reaction of pyrazole with 1-bromo-2-methylnaphthalene gave a mixture of two ligands (MeNap-Pz and NapMe-Pz). Because bromine atom that was bonded to seconder carbon of naphthalene led to halogen migration to methyl group, NapMe-Pz ligand was produced as a by-product. Therefore, it was decided to perform the reaction of pyrazole with 2-(bromomethyl)naphthalene to obtain NapMe-Pz. Analytical results verified the proposed formulas for the MeNap-Pz and NapMe-Pz compounds. NMR, FT-IR, and mass spectra results of these ligands were confirmed by comparison with the structures of synthesized compound previously [28,29]. Copper complexes of the MeNap-Pz and NapMe-Pz ligands were synthesized for the first time and characterized with NMR, FT-IR, mass spectra, UV-Vis, and ESR. Although MeNap-Pz and NapMe-Pz ligands were soluble in organic polar solvents, they were sparingly soluble in EtOH. Complexes were soluble in organic solvents such as EtOH, MeOH, acetonitrile, and acetone, while they were insoluble in water and nonpolar organic solvents such as diethyl ether, hexane, and petroleum ether. Although NapMe-Pz ligand had poor solubility in EtOH, complex of [Cu(NapMe-Pz)_2_(NO_3_)_2_].H_2_O was soluble in EtOH.

The infrared data of pyrazole ligands and their Cu complexes (Figures S1–S4) are summarized in Table 2. The IR spectra of pyrazole ligands show weak absorption bands in the range of 3130–3047 cm^–1 ^indicating the presence of the aromatic C–H stretching vibrations. Absorption bands at 2970–2922 cm^–1^ should be assigned to the stretching vibrations of the C–H (-CH_3_, -CH_2_). Another characteristic pyrazole bands in the range of 1737–1633 cm^–1 ^(C=N), 1600–1500 cm^–1 ^(C=C), 1370 cm^–1 ^(C-N), and 1047–1040 cm^–1 ^(N-N) are attributed to the aromatic skeleton stretching vibration of the pyrazolyl and naphthyl rings [30,38]. The IR spectra data of the complexes [Cu(MeNap-Pz)_2_(NO_3_)]NO_3_.2H_2_O and [Cu(NapMe-Pz)_2_(NO_3_)_2_].H_2_O showed lattice water absorbs at 3545–3409 cm^–1^ [39] and showed stretching vibrations of aromatic C–H and aliphatic C–H (-CH_3_, -CH_2_) in the range of 3128–3050 cm^–1 ^and 2972–2916 cm^–1^, respectively. The shift of the C=N, C=C, C-N, and N-N of the pyrazole and naphthaline rings bands frequencies compared to that of the free ligands bonds showed the interaction between Cu(II) and pyrazole ligands [30]. The characteristic bands of NO_3_^-^ anion are shown at 1483–1480 cm^–1^ for asymmetric stretching vibrations (u_5_), 1277–1275 cm^–1^ for symmetric stretching vibrations (u_1_) and 1011–1010 cm^–1 ^for (N=O) stretching vibrations (u_2_). The coordination modes of the nitrate ions are important in clarifying the geometric structures of the synthesized copper nitrate complexes. The nitrate ion can be bonded to the metal in different fashions, such as monodentate, bidentate, or chelated bridges in the copper(II) nitrate complexes. Large splitting of stretching vibrations of N–O bonds in a NO_3_^- ^ion (u_5_-u_1_>160) confirms the presence of bidentate nitrate ions of complexes [39–41]. The presence of coordinated water molecules in the complexes is indicated by a broad band in the region 3545–3409 cm^–1^ [42]. IR spectral data results indicate that naphthyl-pyrazole ligands and their Cu(II) complexes were obtained.

**Table 2:** The important infrared frequencies (in cm–1) of naphthyl-pyrazole ligands and Cu(II) complexes.

	MeNap-Pz	NapMe-Pz	[Cu(MeNap-Pz)2(NO3)]NO3.2H2O	[Cu(NapMe-Pz)2(NO3)2].H2O
u(O-H)			3545	3409
u(C-H)ar	3127–3047	3130–3049	3128–3054	3132–3050
u(C-H)al	2922	2970	2916	2972–2947
u(C=N)	1633	1737	1635	1633
u(C=C)	1599–1507	1600–1509	1601–1577	1602
u(C-N)	1370	1370	1373	1396
u(N-N)	1040	1047	1062	1049
u(N-O, NO3)			1480 (u5)1277 (u1)1011 (u2)	1483 (u5)1275 (u1)1010 (u2)

^1^H and ^13^C NMR spectra of ligands (Figures S5–S8) were recorded in CDCl_3_. In ^1^H NMR, MeNap-Pz and NapMe-Pz ligands showed the aromatic protons at d = 7.1–7.8 ppm and a typical proton signal (H_4_) at d = 6.5 ppm, (CH_3_) at d = 2.2 ppm for MeNap-Pz and (H_4_) at d = 6.2 ppm, (CH_2_) at d = 5.4 ppm for NapMe-Pz. In ^13^C-NMR (CDCl_3_) spectra of ligands showed the aromatic carbons for naphthalene at d = 134–122.5 ppm. Overlapping occurrences are observed because some of the aromatic ring carbons are symmetrical. The signals were observed at d = 140.4 ppm and d = 139.5 ppm for pyrazole (C_3_) and (C_5_) carbons and specific signal for pyrazole at d = 106 ppm for (C_4_) carbon for both of ligands. The signals of CH_2_ carbon of NapMe-Pz and CH_3_ carbon of MeNap-Pz were observed at d = 56 ppm and d = 17.5 ppm, respectively. NMR data confirms the proposed structure of the ligands [30,43,44]. NMR spectra of both complexes were measured, but the spectrum of [Cu(NapMe-Pz)_2_(NO_3_)_2_].H_2_O complex was broad due to the paramagnetic property of copper and the signals could not be observed clearly. Although the spectrum of [Cu(MeNap-Pz)_2_(NO_3_)]NO_3_.H_2_O complex was observed considerably broad compared to spectrum of ligand, its structure was fully clarified (Figures S9 and S10). Aromatic ring protons (C-H) was observed at d = 9.9–7.9 ppm and pyrazole protons (H_4_) at d = 6.5 ppm and CH_3_ protons at d = 2.2 ppm in the ^1^H NMR (DMSO-d_6_) spectra of complex. Aromatic carbons were observed at d = 134–122.5 ppm for naphthaline and pyrazole carbons at d = 140.7 ppm (C_3_) and d = 135.4 ppm (C_5_) in the ^13^C NMR (DMSO-d_6_) spectrum of complex. Chemical shift of the pyrazole carbon (C_5_) moved to high frequency on the complex. In the HETCOR-NMR spectrum of the [Cu(MeNap-Pz)_2_(NO_3_)]NO_3_.2H_2_O complex (Figure S11), it is seen that the numbers and signals of the hydrogen atoms and the carbon atoms in the aromatic ring are matched. Moreover, the pyrazole C_4_ carbon at 106.9 ppm matched the H_4_ proton at 6.5 ppm and the CH_3_ group carbon at 17.5 ppm matched proton signals at 2.0 ppm.

NMR spectra were consistent with the signal values of the ligand and a slight shift was observed especially in ^1^H-NMR values due to the binding to the metal. It was also confirmed by HETCOR-NMR that the number of hydrogen atoms attached to the aromatic ring was the same as the ligand, so the ligand was not orthomethalated with copper over the carbon in the aromatic ring.

**Figure 1:**
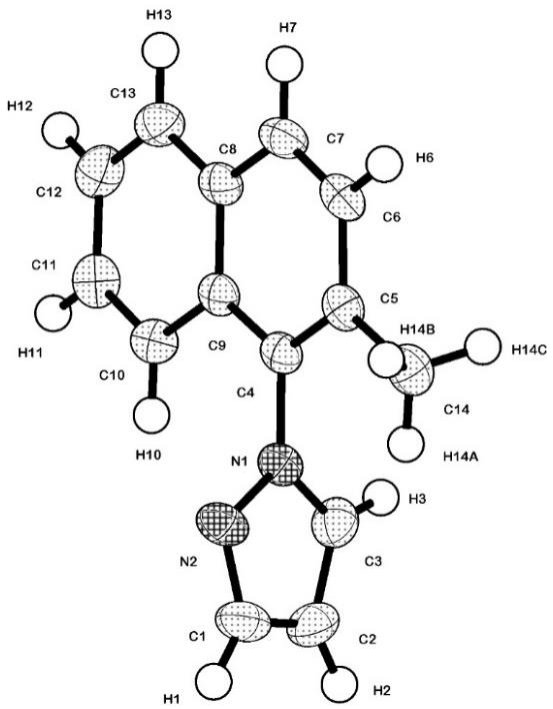
View of MeNap-Pz with the atom-labeling scheme and 50% probability displacement ellipsoids.

MeNap-Pz molecular structure with the atom-numbering scheme is shown in Figure 1, and selected bond lengths and angles are given in Table 3. In the structure of the ligand, all carbon atoms of the naphthyl and pyrazole ring form a good plane, and the average of C–C distances and C–C–C angles from the ring are consistent with those reported in the literature [45]. The naphthyl and pyrazole rings are almost orientated perpendicular to each other at an angle of 80.62(13)°.

**Table 3:** Bond lengths [Å] and angles [°] for MeNap-Pz.

Bond lengths	(Å)	Angles	(°)
N(1)-C(3)	1.342(3)	C(3)-N(1)-N(2)	111.88(18)
N(1)-N(2)	1.362(2)	C(1)-N(2)-N(1)	103.77(19)
N(1)-C(4)	1.435(3)	C(3)-N(1)-C(4)	127.22(18)
N(2)-C(1)	1.332(3)	N(2)-C(1)-C(2)	112.2(2)
C(1)-C(2)	1.388(4)	C(3)-C(2)-C(1)	104.8(2)
C(2)-C(3)	1.363(3)	N(1)-C(3)-C(2)	107.4(2)
C(5)-C(14)	1.505(3)	C(6)-C(5)-C(14)	119.71(19)

The TG and DTG curves for the complexes are shown in Figures S12 and S13. Table 4 lists the results of the thermal studies of these complexes. The decomposition of the complex [Cu(MeNap-Pz)_2_(NO_3_)]NO_3_.2H_2_O in CuO occurs in three steps. In the first stage (99.4 °C), loss of two molecules water 5.91% (Calcd. 5.6%) of the total weight is observed. In the next stage of decomposition (220 °C), there is loss of 66.15% (Calcd. 65%) of the total weight attributed to the elimination of MeNap-Pz ligand [30]. The complex then showed a third weight loss in the range of 300–776 °C, a loss of 27.23% (Calcd. 29.37%), which corresponds to loss of NO_3_^-^ and residue of CuO [46]. The TG–DTG curves for complex Cu(NapMe-Pz)_2_(NO_3_)_2_].H_2_O (Figure S12) indicated that in the first stage (132.8 °C), there is loss of a water molecule 2.2% (Calcd. 2.89%) of the total weight. In the second stage of decomposition (242.4 °C), there is loss of 63.69 (calcd. 66.87%) of total weight attributed to the elimination of NapMe-Pz ligand [30]. In the third stage, the loss of NO_3_^- ^anions 22.59% (calcd. 19.93%) is in the range of 300–600 °C [46]. The final decomposition residue was CuO 11.25% (calcd. 10.21%) over the range of 600 °C.

**Table 4:** Thermal analysis data for the complexes.

	[Cu(MeNap-Pz)2(NO3)]NO3.2H2OF.W.: 640 g/mol	[Cu(NapMe-Pz)2(NO3)2].H2OF.W.: 622.09 g/mol
DT(°C)	50–100100–776776–800	100–200100–300300–600
Dm(Exp)(%)	5.9166.1527.23	2.263.6922.5911.25
Dm(Calcd.)(%)	5.636529.37	2.8966.8719.9310.31
Decomp. Steps	CuO	CuO
DTG(peak)(°C)	99.4220776	132.8242.4

Molecular ion peak of MeNap-Pz was determined as 208 in Figure S14. The peaks obtained by removal of –CH_3_ and HCN from pyrazole of the ligand were observed at 192 and 180 [M-HCN] (100%), respectively. In Figure S15, NapMe-Pz ligand molecular ion peak is [M-] 207 and the peak obtained by removal of pyrazole from the ligand was observed at 141 [M-Pz] (100%). Pyrazole bonded to methylene bridge (-CH_2_-) was removed easily than that bonded to aromatic naphtyl group. The LC-MS/MS spectra of these complexes (Figures S16 and S17) are summarized in Table 5. Molecular ion peaks for [Cu(NapMe-Pz)_2_(NO_3_)_2_].H_2_O (F.W.: 622 g/mol): [Cu(NapMe-Pz)_2_(NO_3_)]^+^ , 538, [Cu(NapMe-Pz)_2_]^+2 ,^479, and peak of NapMe-Pz (M.W.: 208 g/mol) ligand was observed at 209. Molecular ion peaks for [Cu(MeNap-Pz)_2_(NO_3_)]NO_3_.2H_2_O (F.W.: 640 g/mol): [Cu(MeNap-Pz)_2_(NO_3_)]^+^ (F.W.: 542 g/mol), [Cu(MeNap-Pz)_2_]^+2^ (F.W.: 480 g/mol), [MeNap-Pz] (M..W: 208 g/mol) were seen at 541, 481, 209, respectively.

**Table 5:** Physical and analytical data for the complexes.

Complex	Color	Yield%	MassLC-MS/MS(Calc.)	ConductivityΩ–1cm2mol–1	Magnetic SusceptibilityBM	% CuExp. (Calc.)
[Cu(MeNap-Pz)2(NO3)](NO3).2H2O	Green	52	633(640)	129	1.75	11.90(10.21)
[Cu(NapMe-Pz)2(NO3)2].H2O	Green	40	623(622.9)	13	2.27	9.34(9.92)

Analytical and spectroscopic data of the synthesized complexes are given in Table 5. Complexes conductivity measured in 1 × 10^-3^ M methanol. The reported values for 1:1 and 1:2 electrolytes in methanol are 80–115 and 160–220 Ω^–1^cm^2^mol^–1 ^[47]. Complexes conductivity in methanol ranged between 13 and 129, which is consistent with [Cu(NapMe-Pz)_2_(NO_3_)_2_].H_2_O is nonionic and [Cu(MeNap-Pz)_2_(NO_3_)](NO_3_).2H_2_O is electrolyte 1:1.

The experimental magnetic moment of the complex was found to be 1.75 and 2.27 B.M. The results showed that both complexes are paramagnetic and Cu is coordinated to ligands as mononuclear. The value of [Cu(NapMe-Pz)_2_(NO_3_)_2_].H_2_O complex is in accordance with their distorted octahedral configurations [48]. According to the ICP-OES results, wt % Cu of the complexes were found and compared well with their theoretical values.

The electronic spectra of ligands and their copper(II) complexes were recorded and spectral data are given in Table S1 and Figures S18 and S19. Compared with the p-p*transition of the ligands with complexes, the slight movement of the absorption band of the complexes indicates the charge transfer from ligand to metal or metal to ligand [49]. The presence of strong absorption bands at 225 and 223 nm in the complexes indicates that there are ligand-centered bands. In the complexes, additional bands caused by d-d* transitions of Cu^2+ ^were not seen. The reason why additional bands do not clearly appear in the complexes is thought to be that the p-p* transitions of the ligands overlap with other transitions by giving strong absorption bands.

The ESR spectra of copper(II) complexes in powder samples (Figures S20 and S21 and Table S2) were recorded at room temperature. 

The ESR spectrum of [Cu(MeNap-Pz)_2_(NO_3_)]NO_3_.2H_2_O complex shows g_11 _(2.21) > g_^_(2.05) value is characteristic of an axially elongated square-planar geometry. If the copper content in the compound is high, the hyperfine line from either ^63^Cu or ^65^Cu cannot be observed. The ESR results are g_11 _> g_^_> 2.00 and this is a characteristic feature of dx^2^-y^2^ ground state [50,51]. Considering the ESR spectra, square planar geometry for this complex was suggested and copper(II) ion in this this complex in a tetragonal field (D4h) symmetry [52]. ESR results give the geometric parameter, G which is defined (Eq. 1) as

 (Eq.1)

G value of the complex 3.9 < 4, suggesting unit cell contains magnetically equivalent ions and ligands are strong field in character [51,52]. According to our results, the G value is less than 4, which means that significant exchange coupling is present [50]. The ESR signals of [Cu(NapMe-Pz)_2_(NO_3_)_2_].H_2_O complex shows well-resolved four line hyperfine splits because copper has a nuclear spin of (I = 3/2) which couples with the electron spin [42]. The observed spectral parameter of complex A_11 _(157) > A_^_(99) and g_11 _(2.35) > g_^_(2.03) indicates that the complex exerts an octahedral geometry [53]. The ESR results are g_11 _> g_^_> 2.00 and this is indicative of a d_x2-y2 _ground state. G value of the complex 10.6 > 4, indicating that the exchange coupling effects are not operative in the present system. The reason is that large volume of ligands can be considered to reduce the exchange interactions between Cu(II) centers [54]. If G is greater than 4, local tetragonal axes are aligned parallel or only slightly misaligned [50]. 

The antibacterial results are presented in Tables 6 and 7 and Figure 2. Gentamycine was used as reference for antibacterial studies. In vitro antibacterial activity data reveals that the antibacterial activity of copper(II) complexes is higher than that of the ligands. As can be seen from Table 6, the greatest effect occurred on gram-positive bacteria *S. aureus* and *B. cereus* . All compounds display least activity against gram-negative bacteria *P. aeruginosa* and *K. pneumoniae* . While the MeNap-Pz ligand shows slight activity against *S. aureus* , the NapMe-Pz ligand is inactive towards all bacteria. While [Cu(NapMe-Pz)_2_(NO_3_)_2_].H_2_O complex shows moderate activity against gram-positive bacteria, [Cu(MeNap-Pz)_2_(NO_3_)]NO_3_.2H_2_O complex shows significant activity against all bacteria especially the gram-positive bacteria.

**Table 6:** In vitro antimicrobial activity by using the agar diffusion method of tested the compounds.

Microorganism inhibition zone diameter mm (Relative inhibition %)				
Compound	Gram+ bacteria		Gram– bacteria	
	Staphylococcus aureus	Bacillus cereus	Klebsiella pneumoniae	Pseudomonas aeruginosa
NapMe-Pz	0(0)	0(0)	0(0)	0(0)
MeNap-Pz	6(28)	0(0)	0(0)	0(0)
[Cu(NapMe-Pz)2(NO3)2]. H2O	12(57)	10(67)	0(0)	0(0)
[Cu(MeNap-Pz)2(NO3)]NO3. 2H2O	14(67)	13(87)	6(37)	6(43)
Gentamycine (10 µg)	21(100)	15(100)	16(100)	14(100)

**Table 7:** Table 7. Minimum inhibition concentration of compounds.

Compound	Staphylococcus aureus ATCC 29213		Pseudomonas aeruginosaATCC 27853		Klebsiella pneumoniae ATCC 700603		Bacillus cereus ATCC 10876	
	MIC	MBC	MIC	MBC	MIC	MBC	MIC	MBC
NapMe-Pz	x	x	x	x	x	x	x	x
MeNap-Pz	1/2	1/2	x	x	x	x	x	x
[Cu(NapMe-Pz)2(NO3)2].H2O	1/16	1/16	x	x	x	x	1/4	1/4
[Cu(MeNap-Pz)2(NO3)]NO3.2H2O	1/8	1/8	1	1	1	1	x	x

The minimum inhibitory concentration (MIC) data of the compounds are represented in Table 7. Depending on the concentration, the suppression of bacterial growth increases, but the effectiveness has not been observed because some compounds’ concentration remains low. It can be said that NapMe-Pz and MeNap-Pz ligands have no activity on bacteria. Only MeNap-Pz ligand is active (MIC: 45 mg/mL) against *S. aureus* bacteria. Looking at these results of the complexes, while [Cu(MeNap-Pz)_2_(NO_3_)]NO_3_.2H_2_O complex is highly active (MIC: 11.25 mg/mL) against *S. aureus* , it is inactive against *B. cereus* bacteria. On the other hand, it is active (MIC: 90 mg/mL) against *P. aeruginosa* and *K. pneumoniae* bacteria with the same concentration. While [Cu(NapMe-Pz)_2_(NO_3_)_2_].H_2_O complex is very much active (MIC: 5.625 mg/mL) against *S.* *aureus* , it is inactive against *P.* *aeruginosa* and *K. pneumoniae* bacteria.

## 4. Conclusion

Pyrazole derivative ligands, 1-(2-methylnaphthalen-1-yl)-lH-pyrazole (MeNap-Pz) and 1-(naphthalen-2-ylmethyl)-lH-pyrazole (NapMe-Pz), were synthesized in the KOH/DMSO system using Cu catalyst and characterized with FT-IR, NMR, and ESI-MS. The X-ray diffraction structures of the compound MeNap-Pz were determined for the first time. [Cu(NapMe-Pz)_2_(NO_3_)_2_].H_2_O and [Cu(MeNap-Pz)_2_(NO_3_)](NO_3_).2H_2_O complexes were synthesized and characterized using magnetic susceptibility, molar conductivity, FT-IR, NMR, LC MS-MS, ICP-OES, TGA, and ESR spectra. [Cu(NapMe-Pz)_2_(NO_3_)_2_].H_2_O complex is nonionic and [Cu(MeNap-Pz)_2_(NO_3_)](NO_3_).2H_2_O complex is electrolyte 1:1 as a result of conductivity in methanol 13 and 129 Ω^–1^cm^2^mol^–1^, respectively. The ESR spectra of [Cu(MeNap-Pz)_2_(NO_3_)]NO_3_.2H_2_O complex shows an axially elongated square-planar geometry and [Cu(NapMe-Pz)_2_(NO_3_)_2_].H_2_O complex shows an octahedral geometry. 

The synthesized compounds were tested with antibacterial screening. The new Cu(II) complexes show better antibacterial activity than ligands. Although [Cu(MeNap-Pz)_2_(NO_3_)](NO_3_).2H_2_O complex has the highest recorded inhibition zones for antibacterial activities, it has low MIC values. [Cu(NapMe-Pz)_2_(NO_3_)_2_].H_2_O complex has moderate recorded inhibition zones and strong MIC values for gram-positive bacteria, namely *S. aureus* . Therefore, the new Cu(II) complexes may be effective for the further development of biologically active agents.

Appendix A. Supplementary Data
