# Supplementary material for: Synthesis, characterization, and biological evaluation of new copper complexes of naphthyl pyrazole ligands
Source: Turk J Chem. 2021 Jun 30;45(3):634–46. doi: 10.3906/kim-2010-5 (PMC8326489; doi:10.3906/kim-2010-5)
Supplement: Supplementary file 1 — Appendix A. Supplementary Data [file turkjchem-45-634-sup001.pdf]

Supplement

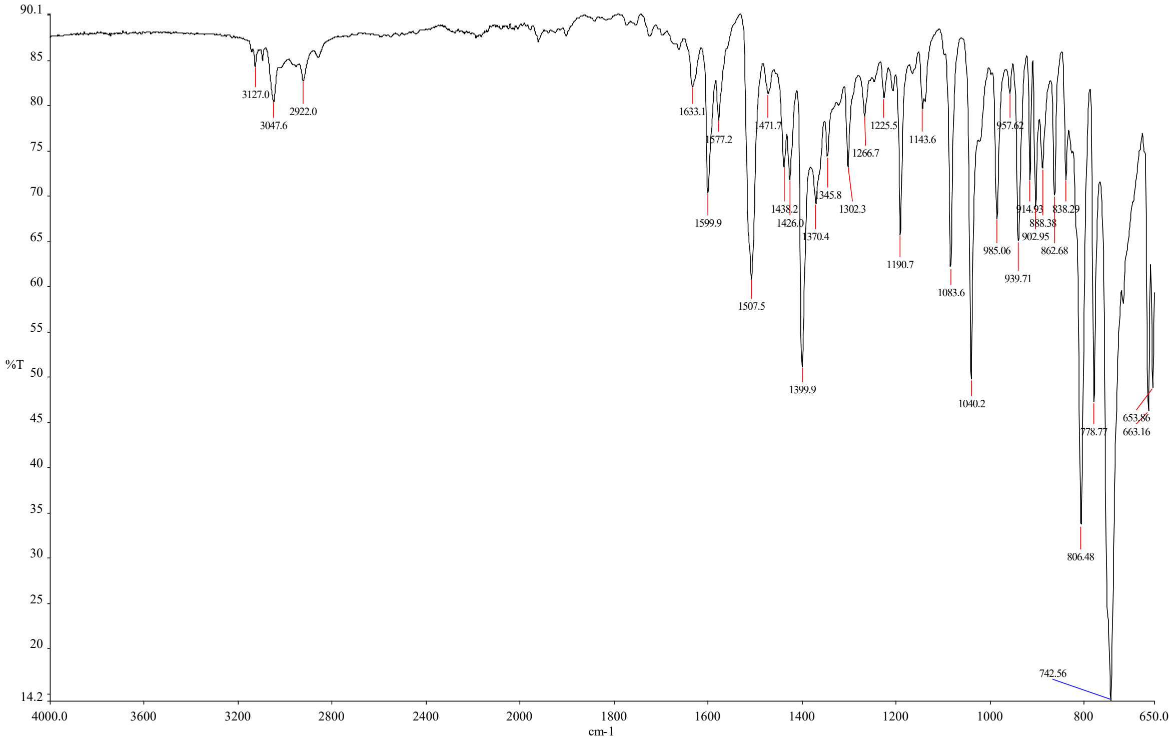

Figure S1. FT-IR spectrum of MeNap-Pz ligand.

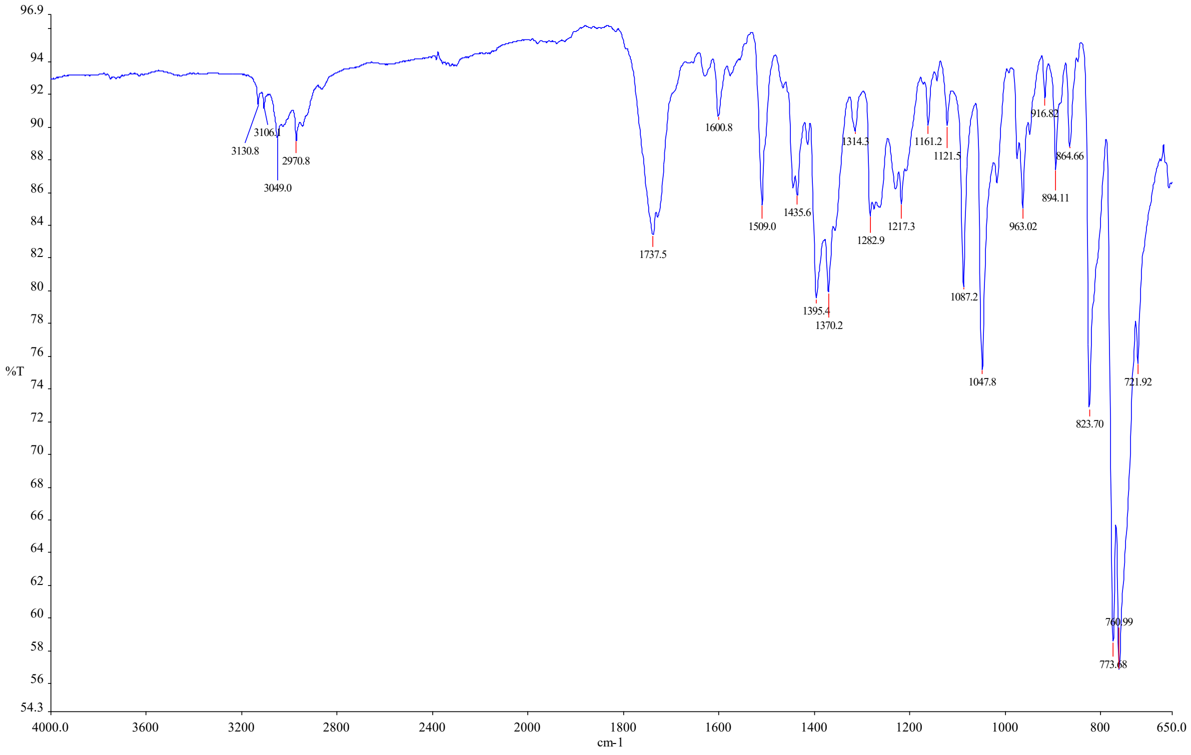

Figure S2. FT-IR spectrum of NapMe-Pz ligand.

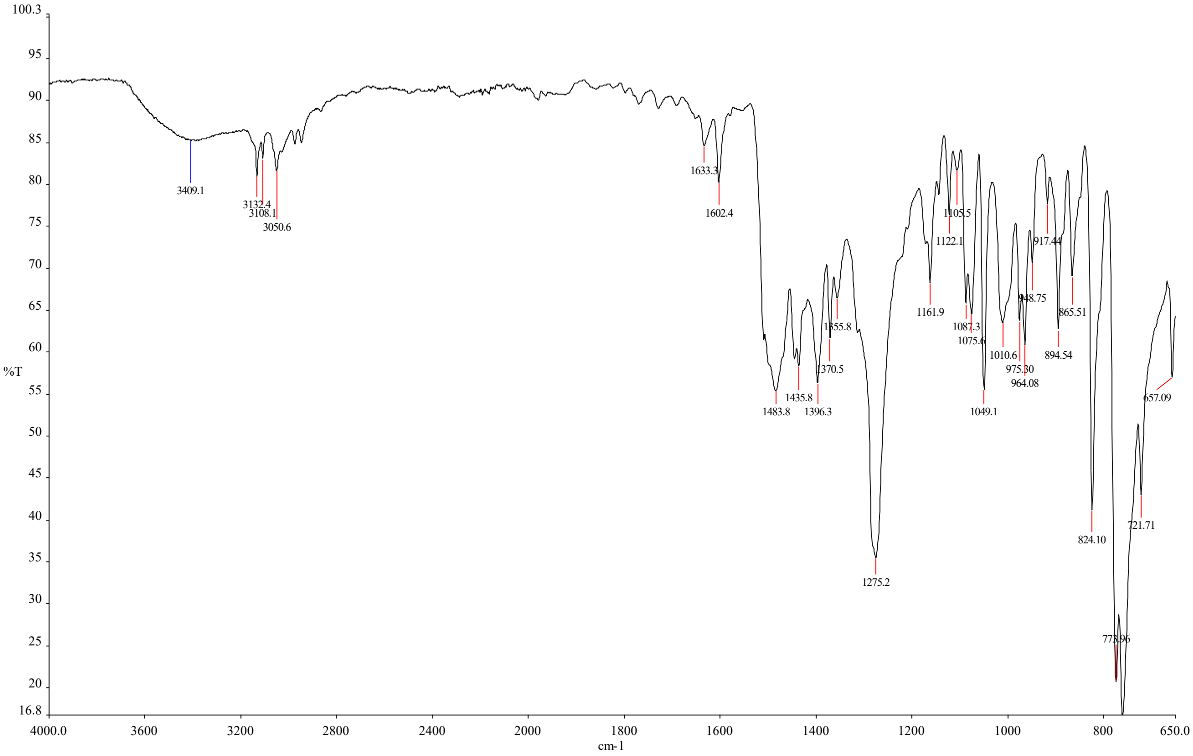

Figure S3. FT-IR spectrum of [Cu(NapMe-Pz)$_2$(NO$_3$)$_2$].H$_2$O complex.

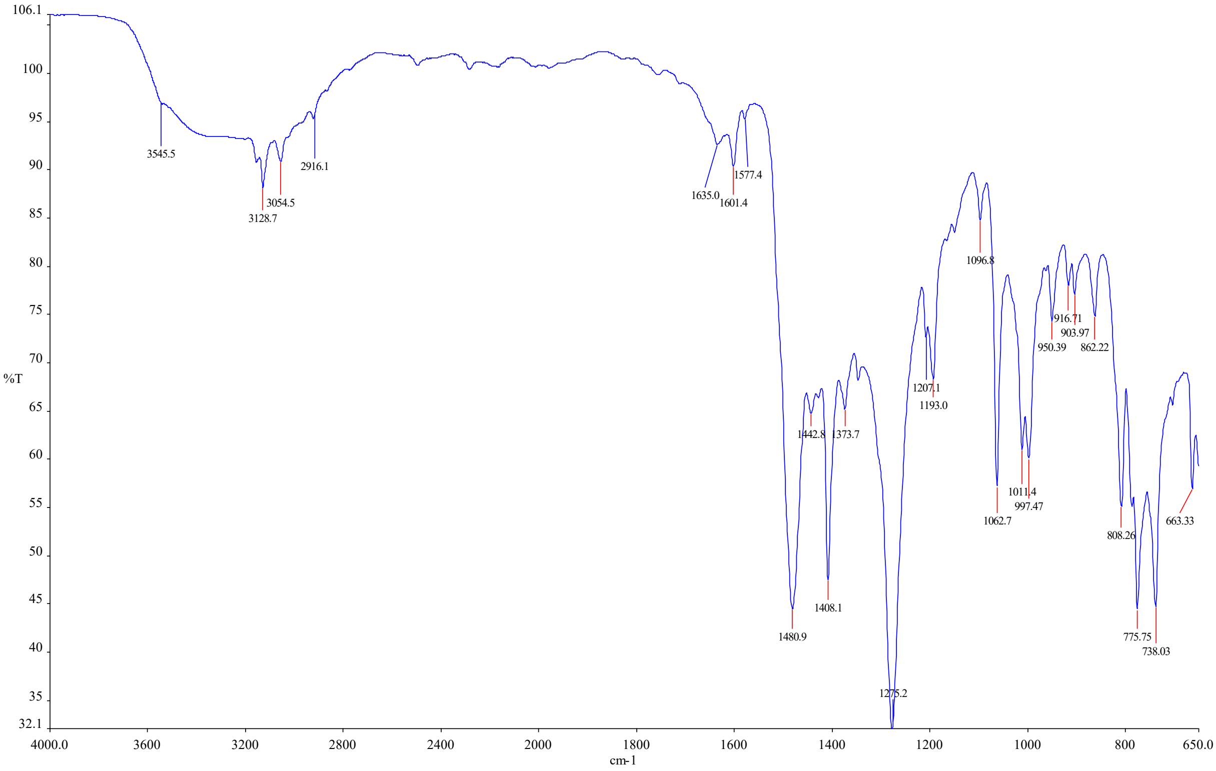

Figure S4. FT-IR spectrum of [Cu(MeNap-Pz)$_2$(NO$_3$)]NO$_3$.2H$_2$O complex.

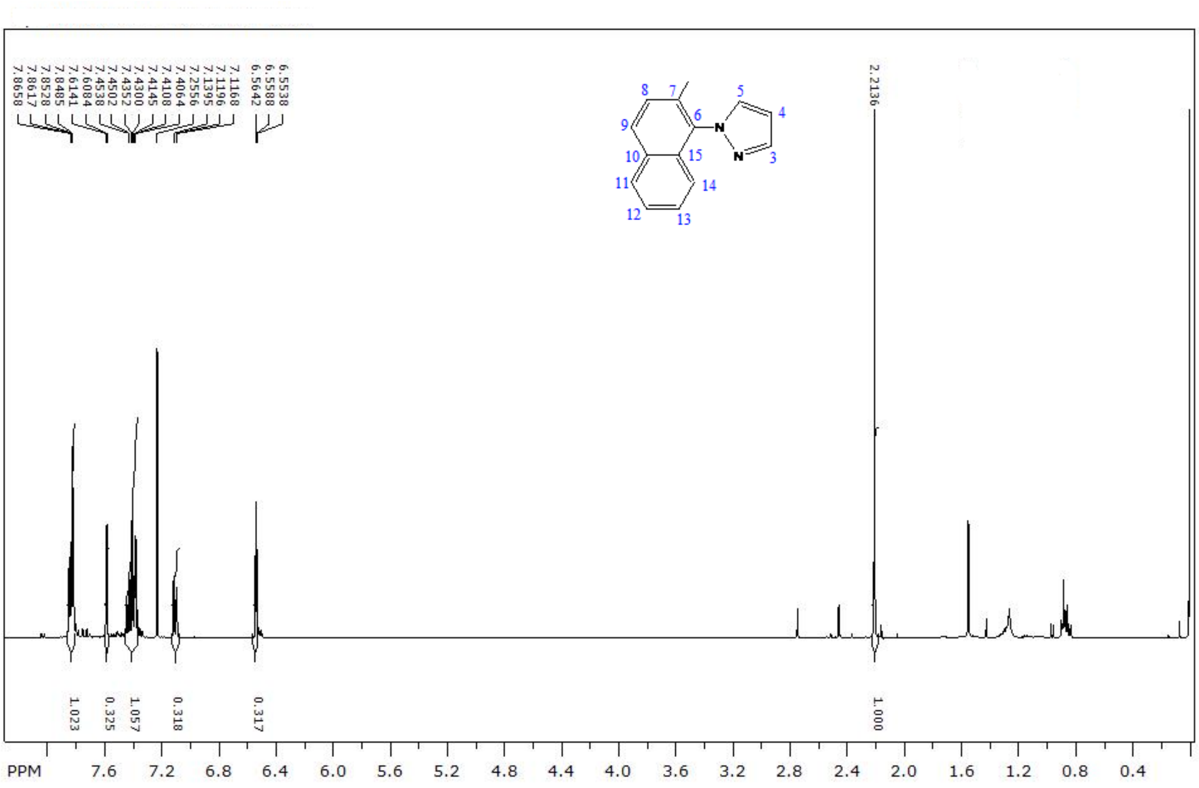

Figure S5. ^{1}H-NMR spectrum of MeNap-Pz ligand recorded in $CDCl_3$ and 400 MHz.

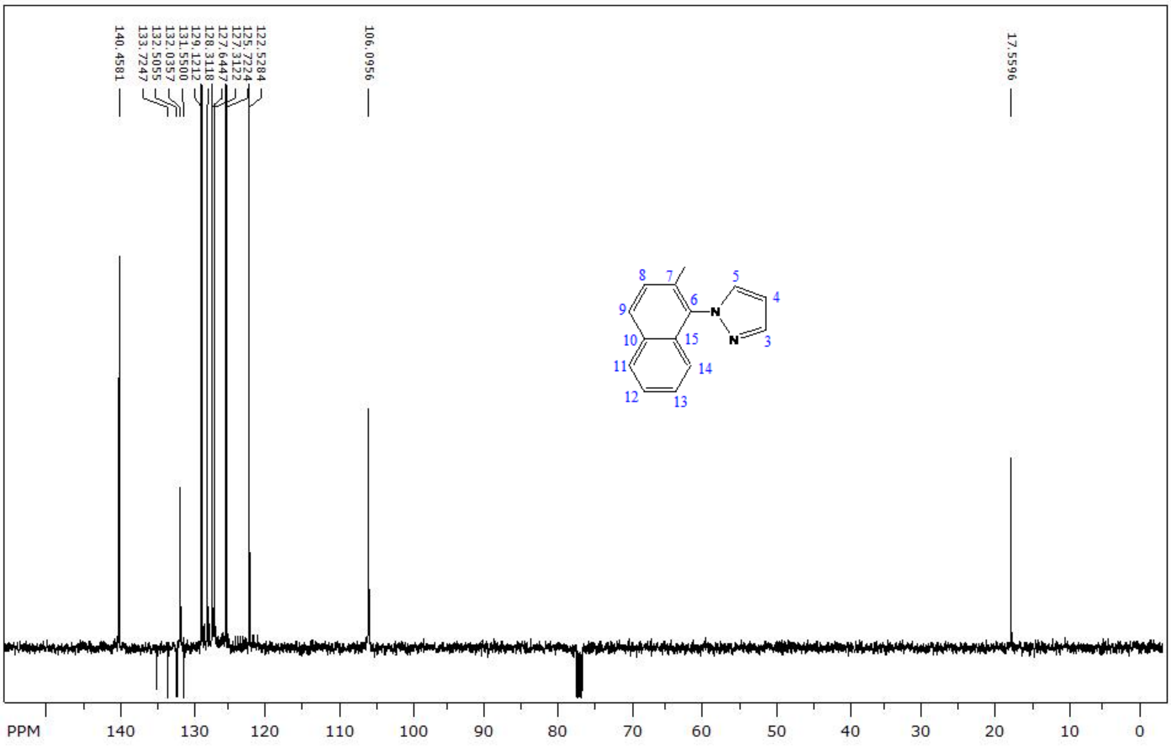

Figure S6. ^{13}C-NMR (ATP) spectrum of MeNap-Pz ligand recorded in $CDCl_3$ and 100.6 MHz.

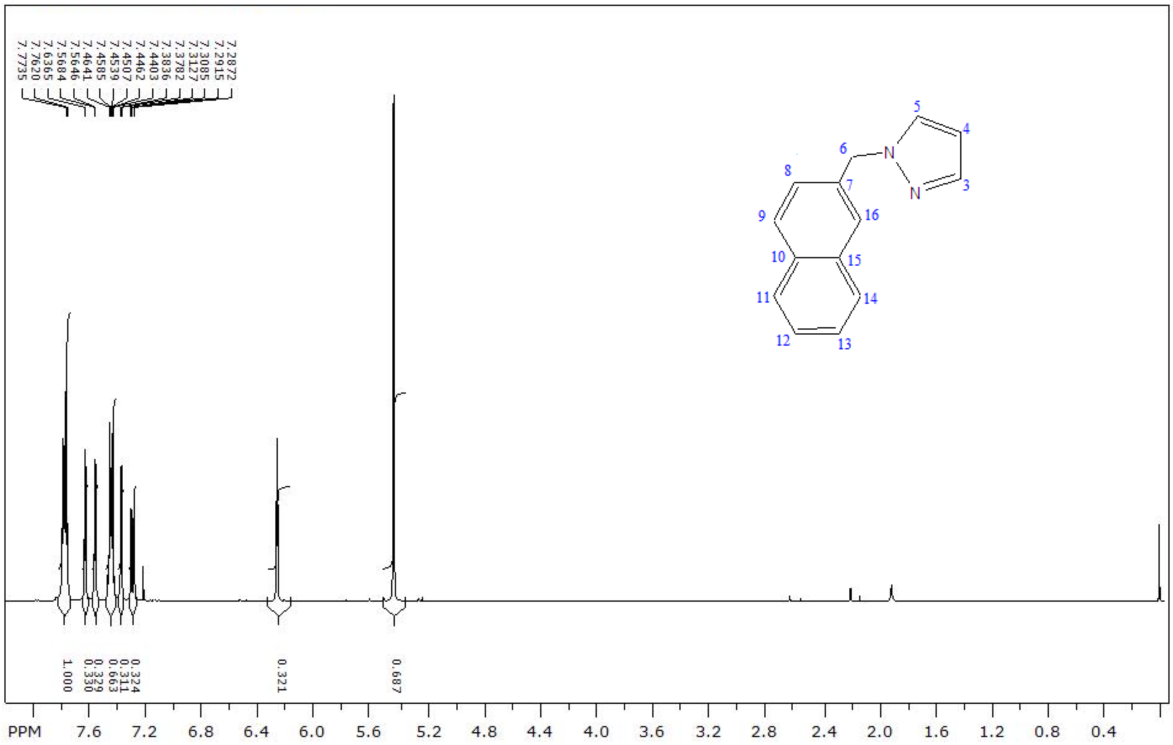

Figure S7. ^{1}H-NMR spectrum of NapMe-Pz ligand recorded in $CDCl_3$ and 400 MHz.

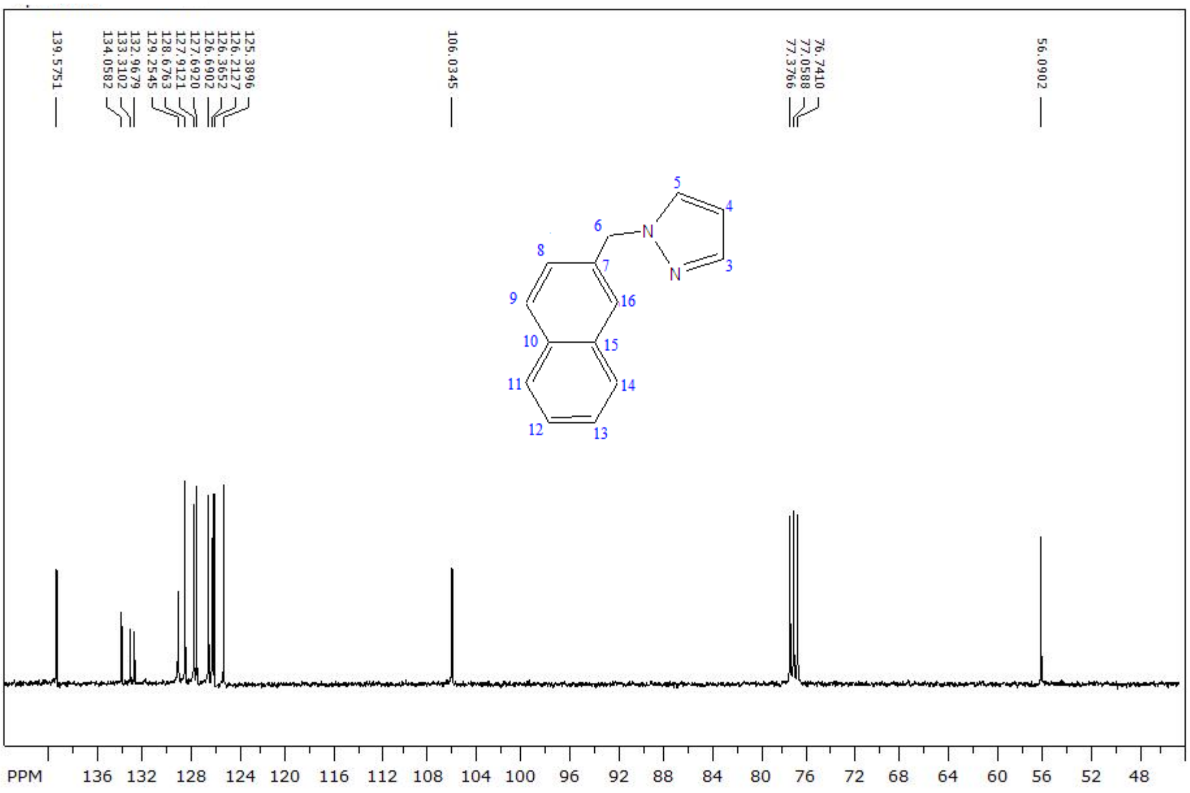

Figure S8. ^{13}C-NMR spectrum of NapMe-Pz ligand recorded in $CDCl_3$ and 100.6 MHz.

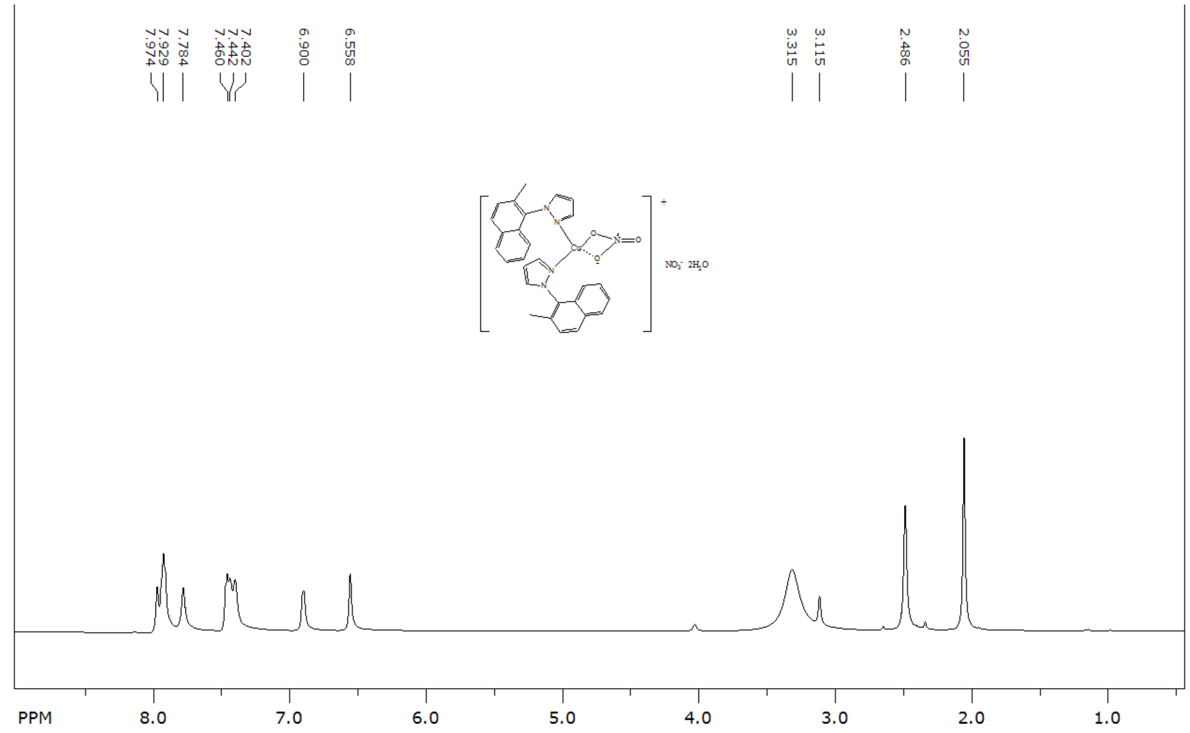

Figure S9. ^{1}H-NMR spectrum of $[Cu(MeNap\text{-}Pz)_2(NO_3)]NO_3.2H_2O$ complex recorded in DMSO-d_6 and 600 MHz.

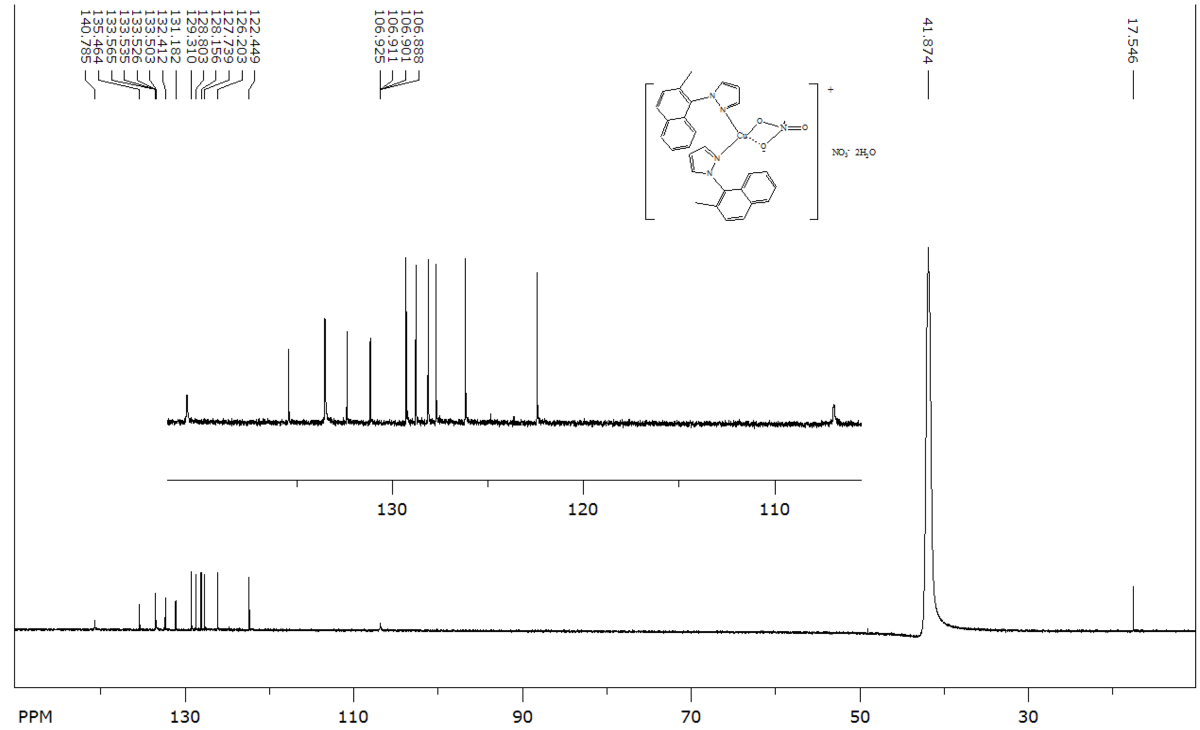

Figure S10. ^{13}C-NMR spectrum of $[Cu(MeNap\text{-}Pz)_2(NO_3)]NO_3.2H_2O$ complex recorded in DMSO-d_6 and 150 MHz.

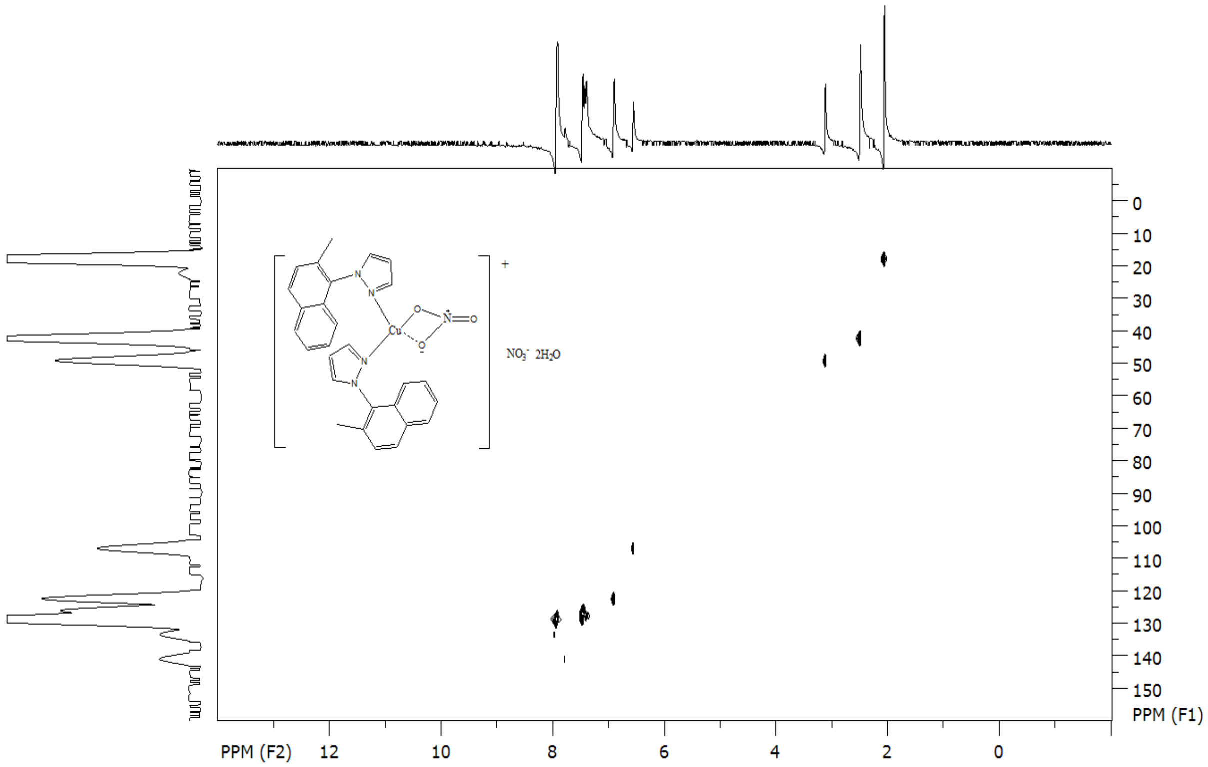

Figure S11. HETCOR-NMR spectrum of [Cu(MeNap-Pz)$_2$(NO$_3$)]NO$_3$.2H$_2$O complex.

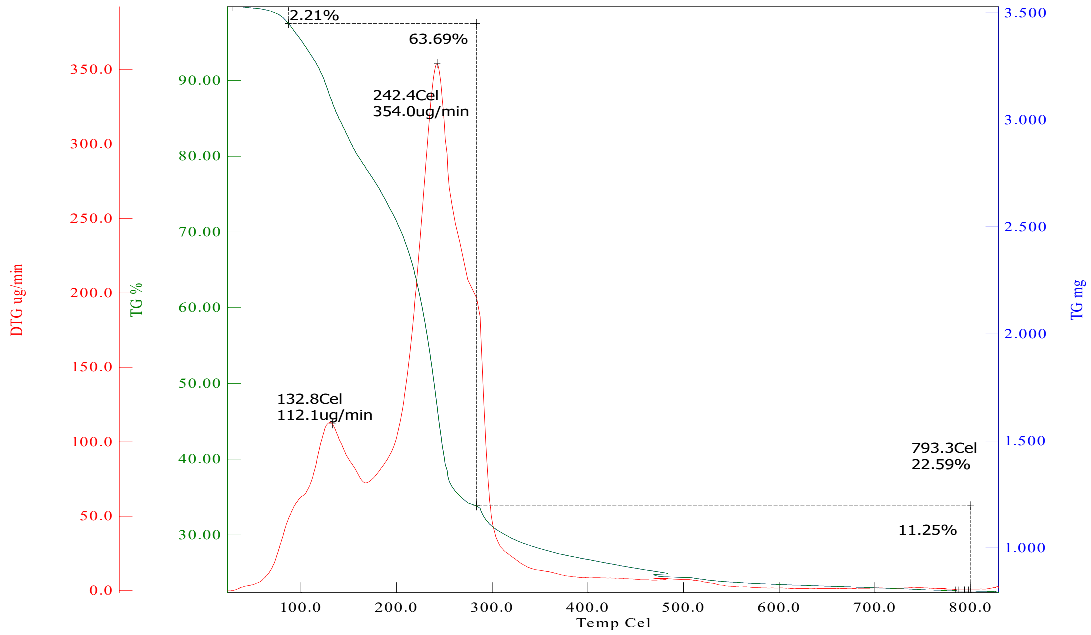

Figure S12. TG-DTG curves of [Cu(NapMe-Pz)$_2$(NO$_3$)$_2$].H$_2$O complex.

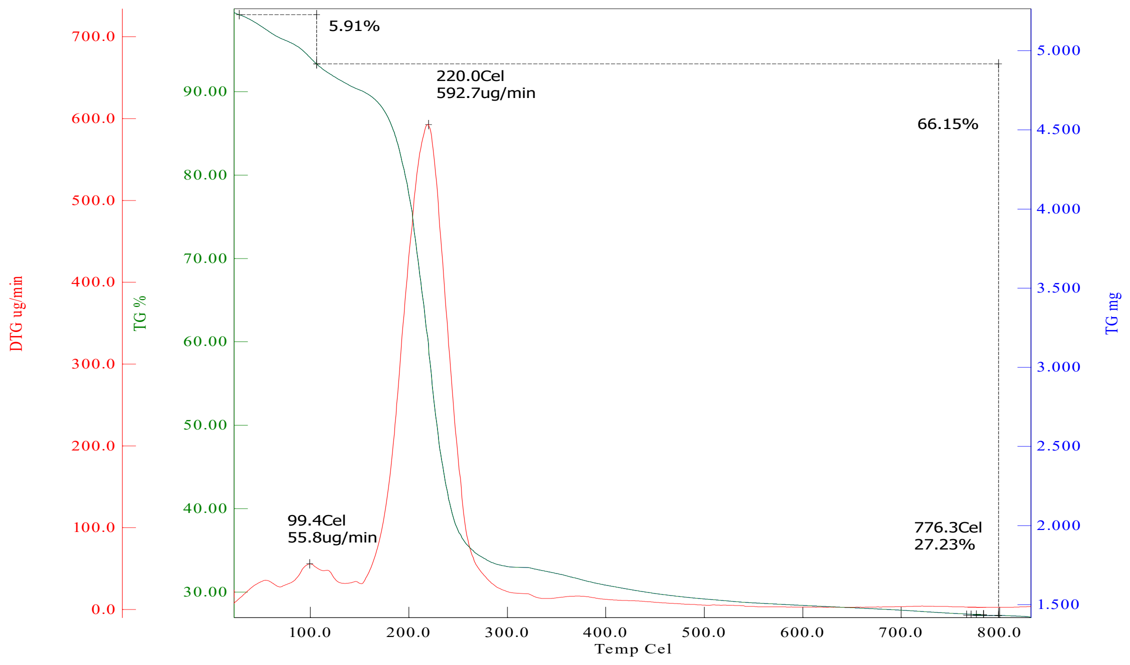

Figure S13. TG-DTG curves of [Cu(MeNap-Pz)$_2$(NO$_3$)]NO$_3$.2H$_2$O complex.

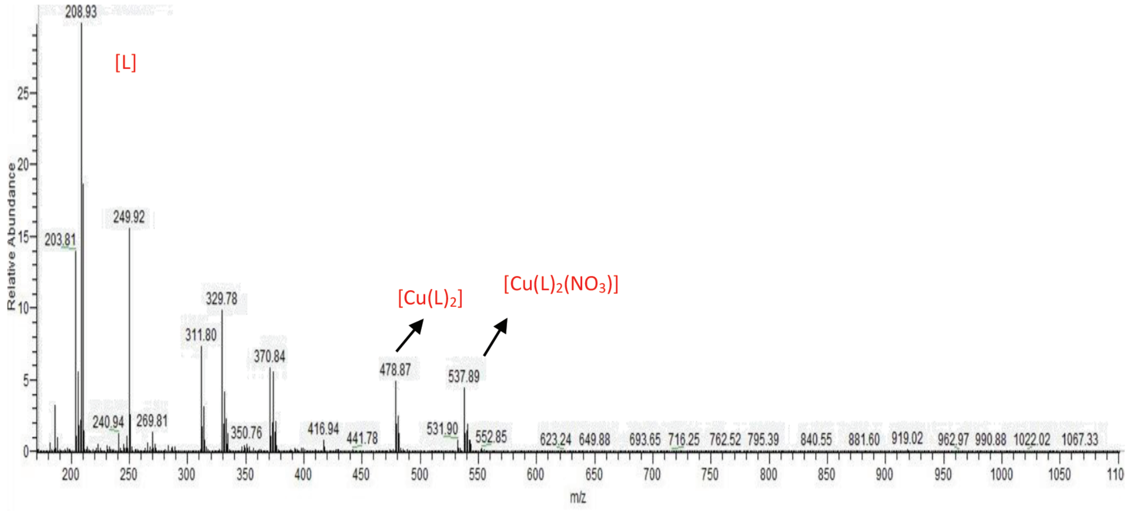

Figure S14. Mass spectrum of MeNap-Pz ligand.

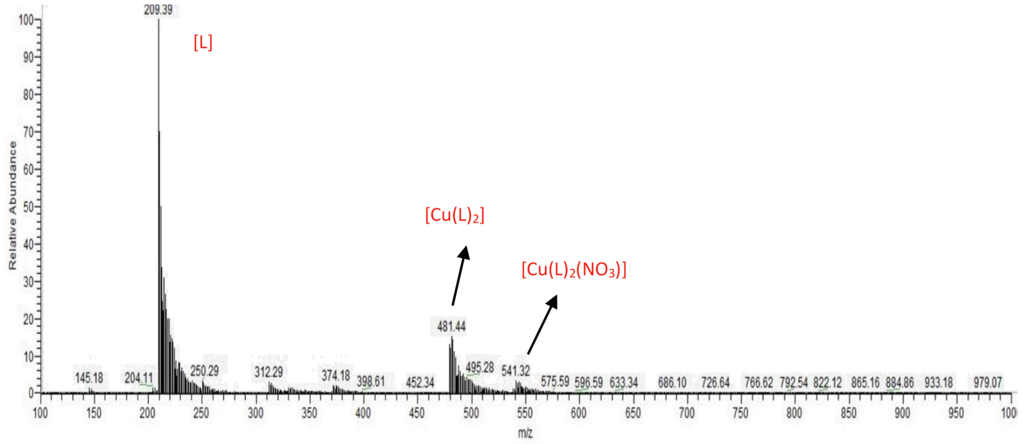

Figure S15. Mass spectrum of NapMe-Pz ligand.

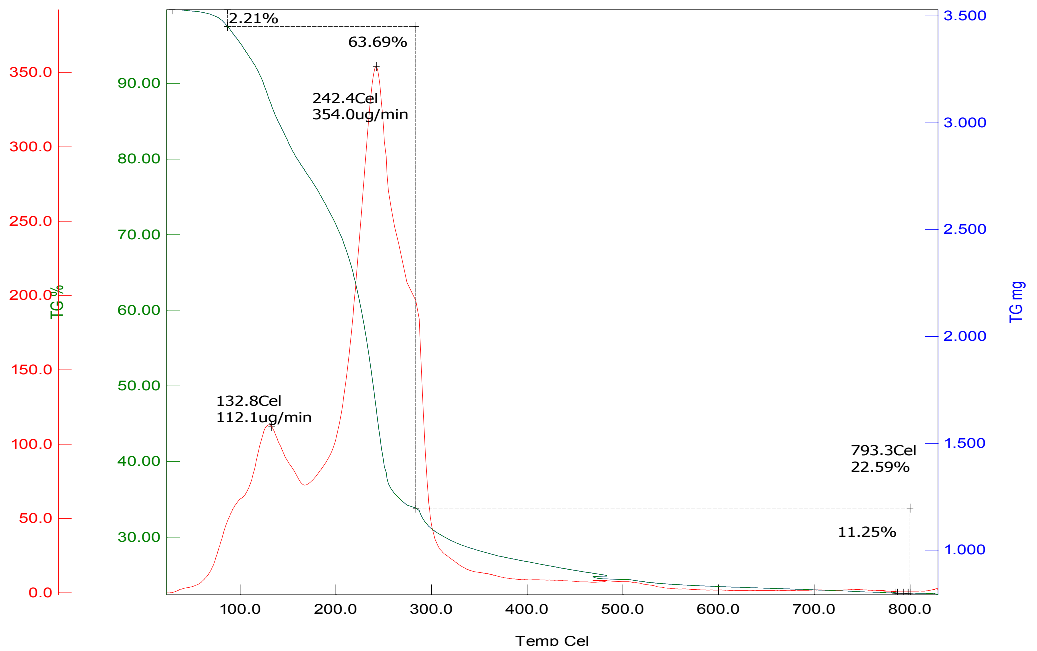

Figure S16. Mass spectrum of [Cu(NapMe-Pz)$_2$(NO$_3$)$_2$].H$_2$O complex.

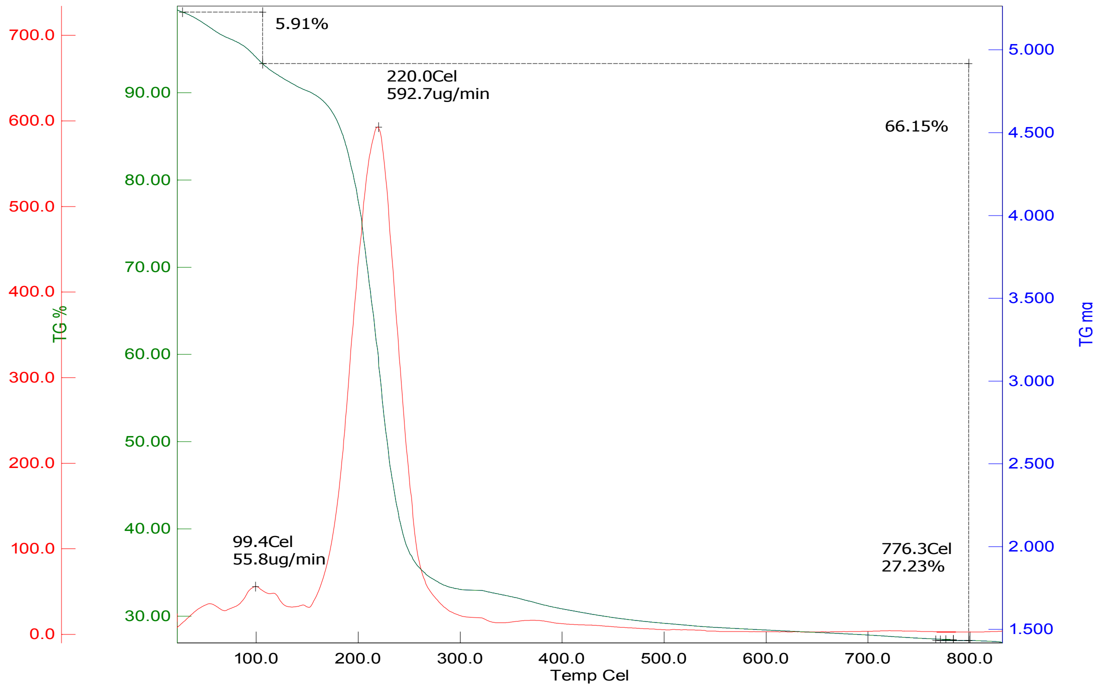

Figure S17. Mass spectrum of [Cu(MeNap-Pz)$_2$(NO$_3$)]NO$_3$.2H$_2$O complex.

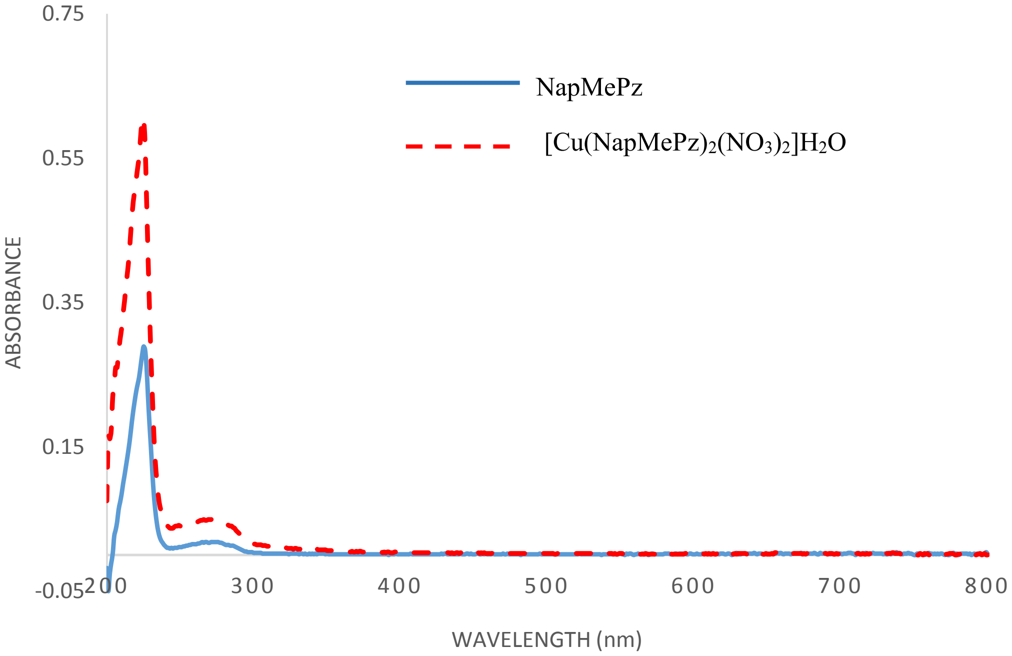

Figure S18. UV-Vis spectrum of NapMe-Pz ligand and its Cu(II) complex.

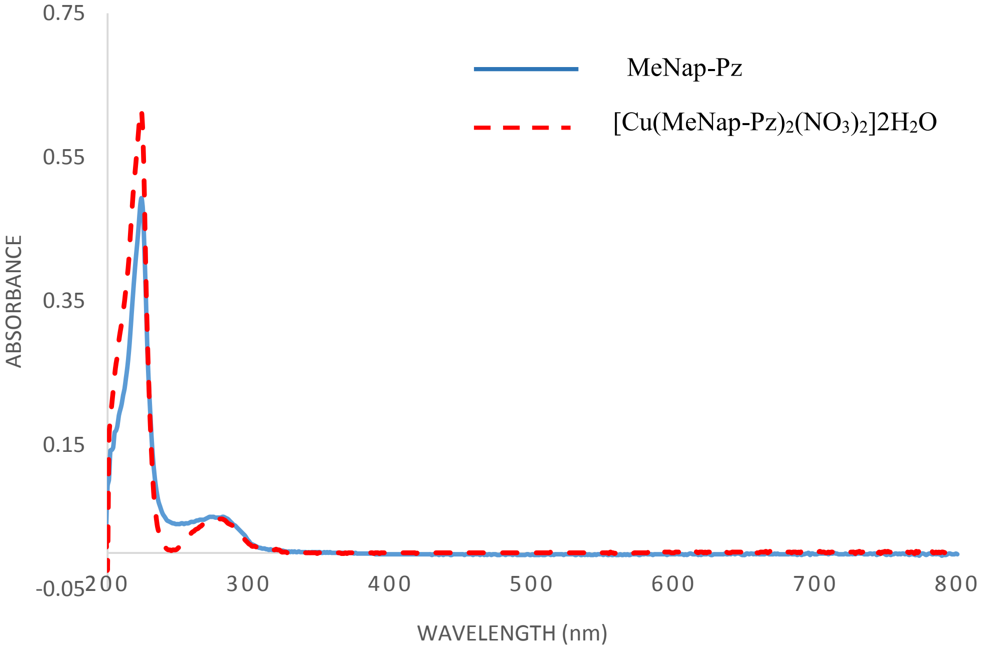

Figure S19. UV-Vis spectrum of MeNap-Pz ligand and its Cu(II) complex.

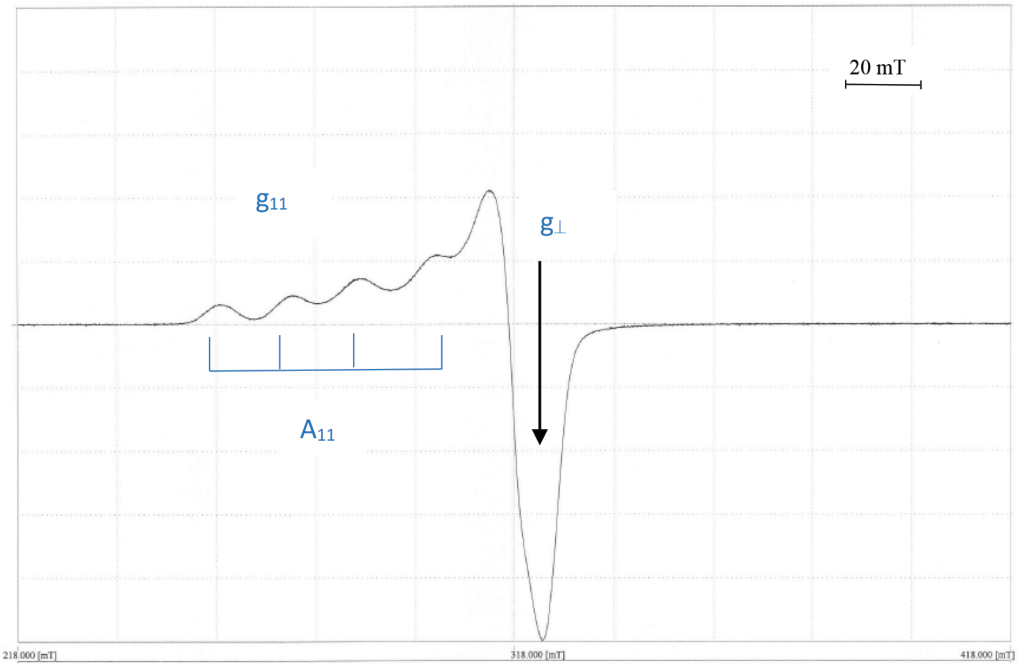

Figure S20. ESR spectrum of [Cu(NapMe-Pz)$_2$(NO$_3$)$_2$].H$_2$O complex.

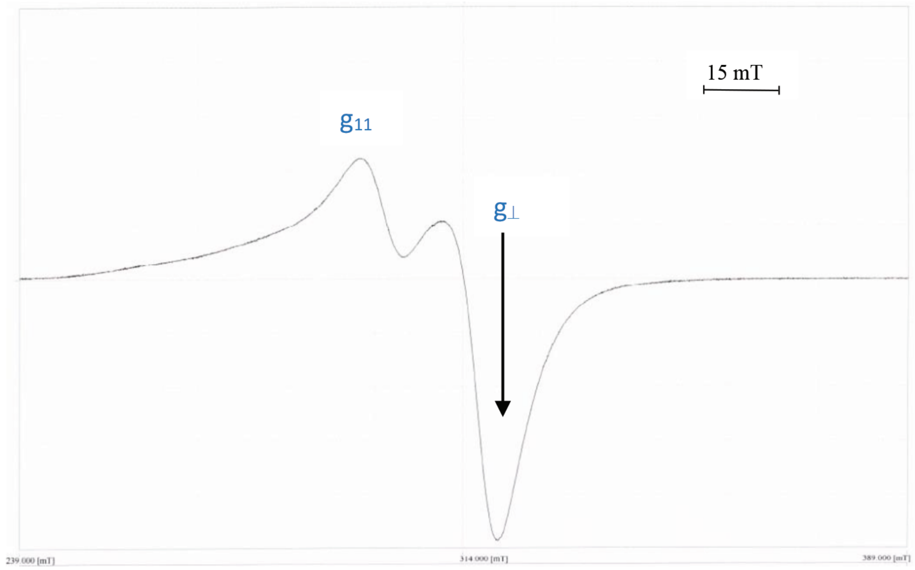

Figure S21. ESR spectrum of [Cu(MeNap-Pz)$_2$(NO$_3$)]NO$_3$.2H$_2$O complex.

Table S1. UV-Vis spectra of ligands and their metal complexes.

Compounds	$\lambda_{max.}$ (nm)	Ɛ ($M^{-1}cm^{-1}$)	Transitions
NapMe-Pz	225<br>273	97306<br>6060	n-π^*<br>π–π^*
[Cu(NapMe-Pz)$_2$(NO_3)$_2$].H_2O	225<br>274	157198<br>13229	n-π^*<br>π–π^*
MeNap-Pz	224<br>281	10000<br>1010	n-π^*<br>π–π^*
[Cu(MeNap-Pz)$_2$(NO_3)]NO_3.2H_2O	223<br>279	130851<br>10000	n-π^*<br>π–π^*

Table S2. ESR parameters for Cu(II) complexes.

Complex	g_{11}	$g_{\perp}$	A_{11} x10^{-4} cm^{-1}	$A_{\perp} \times 10^{-4}$ cm^{-1}	G
[Cu(MeNap-Pz)$_2$(NO_3)]NO_3.2H_2O	2.21	2.05	---	---	3.9
[Cu(NapMe-Pz)$_2$(NO_3)$_2$].H_2O	2.35	2.03	157	99	10.6
